# goSTAG: gene ontology subtrees to tag and annotate genes within a set

**DOI:** 10.1186/s13029-017-0066-1

**Published:** 2017-04-13

**Authors:** Brian D. Bennett, Pierre R. Bushel

**Affiliations:** 1 Integrative Bioinformatics Group, National Institute of Environmental Health Sciences, Research Triangle Park, 27709 NC USA; 2 Biostatistics and Computational Biology Branch, National Institute of Environmental Health Sciences, Research Triangle Park, 27709 NC USA; 3 Microarray and Genome Informatics Group, National Institute of Environmental Health Sciences, 111 T.W. Alexander Drive, Research Triangle Park, 27709 NC USA

**Keywords:** Gene expression, Gene Ontology, GO, Biological themes, Clustering, Over-representation analysis, Subtree, Functional enrichment, Pathway analysis

## Abstract

**Background:**

Over-representation analysis (ORA) detects enrichment of genes within biological categories. Gene Ontology (GO) domains are commonly used for gene/gene-product annotation. When ORA is employed, often times there are hundreds of statistically significant GO terms per gene set. Comparing enriched categories between a large number of analyses and identifying the term within the GO hierarchy with the most connections is challenging. Furthermore, ascertaining biological themes representative of the samples can be highly subjective from the interpretation of the enriched categories.

**Results:**

We developed goSTAG for utilizing GO Subtrees to Tag and Annotate Genes that are part of a set. Given gene lists from microarray, RNA sequencing (RNA-Seq) or other genomic high-throughput technologies, goSTAG performs GO enrichment analysis and clusters the GO terms based on the *p*-values from the significance tests. GO subtrees are constructed for each cluster, and the term that has the most paths to the root within the subtree is used to tag and annotate the cluster as the biological theme. We tested goSTAG on a microarray gene expression data set of samples acquired from the bone marrow of rats exposed to cancer therapeutic drugs to determine whether the combination or the order of administration influenced bone marrow toxicity at the level of gene expression. Several clusters were labeled with GO biological processes (BPs) from the subtrees that are indicative of some of the prominent pathways modulated in bone marrow from animals treated with an oxaliplatin/topotecan combination. In particular, negative regulation of MAP kinase activity was the biological theme exclusively in the cluster associated with enrichment at 6 h after treatment with oxaliplatin followed by control. However, nucleoside triphosphate catabolic process was the GO BP labeled exclusively at 6 h after treatment with topotecan followed by control.

**Conclusions:**

goSTAG converts gene lists from genomic analyses into biological themes by enriching biological categories and constructing GO subtrees from over-represented terms in the clusters. The terms with the most paths to the root in the subtree are used to represent the biological themes. goSTAG is developed in R as a Bioconductor package and is available at https://bioconductor.org/packages/goSTAG

**Electronic supplementary material:**

The online version of this article (doi:10.1186/s13029-017-0066-1) contains supplementary material, which is available to authorized users.

## Background

Gene lists derived from the results of genomic analyses are rich in biological information [1, 2]. For instance, differentially expressed genes (DEGs) from a microarray or RNA-Seq analysis are related functionally in terms of their response to a treatment or condition [3]. Gene lists can vary in size, up to several thousand genes, depending on the robustness of the perturbations or how widely different the conditions are biologically [4]. Having a way to associate biological relatedness between hundreds or thousands of genes systematically is impractical by manually curating the annotation and function of each gene.

Over-representation analysis (ORA) of genes was developed to identify biological themes [5]. Given a Gene Ontology (GO) [6, 7] and an annotation of genes that indicate the categories each one fits into, significance of the over-representation of the genes within the ontological categories is determined by a Fisher’s exact test or modeling according to a hypergeometric distribution [8]. Comparing a small number of enriched biological categories for a few samples is manageable using Venn diagrams or other means of assessing overlaps. However, with hundreds of enriched categories and many samples, the comparisons are laborious. Furthermore, if there are enriched categories that are shared between samples, trying to represent a common theme across them is highly subjective. We developed a tool called goSTAG to use GO Subtrees to Tag and Annotate Genes within a set. goSTAG visualizes the similarities between over-representations by clustering the *p*-values from the statistical tests and labels clusters with the GO term that has the most paths to the root within the subtree generated from all the GO terms in the cluster.

## Implementation

The goSTAG package contains seven functions:

1. loadGeneLists: loads sets of gene symbols for ORA that are in gene matrix transposed (GMT) format or text files in a directory
2. loadGOTerms: provides the assignment of genes to GO terms
3. performGOEnrichment: performs the ORA of the genes enriched within the GO categories and computes *p*-values for the significance based on a hypergeometric distribution
4. performHierarchicalClustering: clusters the enrichment matrix
5. groupClusters: partitions clusters of GO terms according to a distance/dissimilarity threshold of where to cut the dendorgram
6. annotateClusters: creates subtrees from the GO terms in the clusters and labels the clusters according to the GO terms with the most paths back to the root
7. plotHeatmap: generates a figure within the active graphic device illustrating the results of the clustering with the annotated labels and a heat map with colors representative of the extent of enrichment

See the goSTAG vignette for details of the functions, arguments, default settings and for optional user-defined analysis parameters.

The workflow for goSTAG proceeds as follows: First, gene lists are loaded from analyses performed within or outside of R. For convenience, a function is provided for loading gene lists generated outside of R. Then, GO terms are loaded from the biomRt package. Users can specify a particular species (human, mouse, or rat) and a GO subontology (molecular function [MF], biological process [BP], or cellular component [CC]). GO terms that have less than the predefined number of genes associated with them are removed. Next, GO enrichment is performed and *p*-values are calculated. Enriched GO terms are filtered by *p*-value or a method for multiple comparisons such as false discovery rate (FDR) [9], with only the union of all significant GO terms remaining. An enrichment matrix is assembled from the –log10 *p*-values for these remaining GO terms. goSTAG performs hierarchical clustering on the matrix using a choice of distance/dissimilarity measures, grouping algorithms and matrix dimension. Based on clusters with a minimum number of GO terms, goSTAG builds a GO subtree for each cluster. The structure of the GO parent/child relationships is obtained from the GO.db package. The GO term with the largest number of paths to the root of the subtree is selected as the representative GO term for that cluster. Finally, goSTAG creates a figure in the active graphic device of R that contains a heatmap representation of the enrichment and the hierarchical clustering dendrogram, with clusters containing at least the predefined number of GO terms labeled with the name of its representative GO term.

Usage example:

- gene_lists < − loadGeneLists ("gene_lists.gmt")
- go_terms < − loadGOTerms ()
- enrichment_matrix < − performGOEnrichment (gene_lists, go_terms)
- hclust_results < − performHierarchicalClustering (enrichment_matrix)
- clusters < − groupClusters (hclust_results)
- cluster_labels < − annotateClusters (clusters)
- plotHeatmap (enrichment_matrix, hclust_results, clusters, cluster_labels)

## Results

**Fig. 1:**
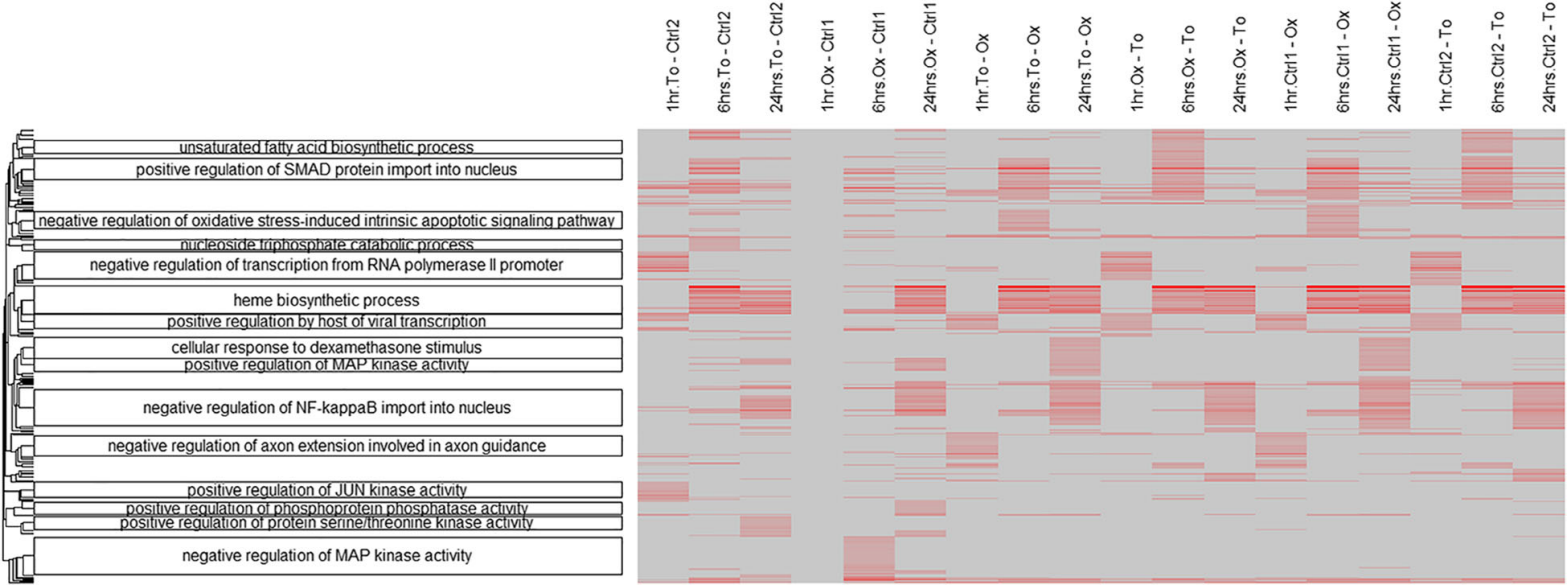
Heat map of GO BPs clustered and labeled with the terms with the most paths to the root. The data used is the –log10 *p*-values from the ORA of the DEG lists. *To*: topotecan, *Ox*: oxaliplatin, *Ctrl*: control. The *x-axis* is the samples, and the *y-axis* is the 762 GO BPs. The more red the intensity, the more significant the enrichment

To demonstrate the utility of goSTAG, we analyzed the DEGs from gene expression analysis (Affymetrix GeneChip Rat Genome 230 2.0 arrays) of samples acquired from the bone marrow of rats exposed to cancer therapeutic drugs (topotecan in combination with oxaliplatin) for 1, 6, or 24 h in order to determine whether the combination or the order of administration influenced bone marrow toxicity at the level of gene expression. Details of the analysis are as previously described [10]. The data are available in the Gene Expression Omnibus (GEO) [11, 12] under accession number GSE63902. The DEG lists (Additional file 1), along with the GO terms from Bioconductor GO.db package v3.4.0 and GO gene associations based on biomaRt package v2.31.4, were fed into goSTAG using default parameters except for the rat species, the distance threshold set at < 0.3 and the minimum number of GO terms in a cluster set at > = 15. The defaults include only considering BP GO terms and requiring at least 5 genes within a GO category. There were 762 BPs significant from the union of all the lists. As shown in Fig. 1, the more red the intensity of the heat map, the more significant the enrichment of the GO BPs. Fifteen clusters of GO BPs are labeled with the term with the largest number of paths to the root in each. Negative regulation of MAP kinase activity (GO:0043407) was the GO BP labeled exclusively in the cluster associated with enrichment at 6 h after treatment with oxaliplatin followed by control. However, nucleoside triphosphate catabolic process (GO:0009143) was the GO BP labeled exclusively in the cluster associated with enrichment at 6 h after treatment with topotecan followed by control.

## Conclusions

goSTAG performs ORA on gene lists from genomic analyses, clusters the enriched biological categories and constructs GO subtrees from over-represented terms in the clusters revealing biological themes representative of the underlying biology. Using goSTAG on microarray gene expression data from the bone marrow of rats exposed to a combination of cancer therapeutics, we were able to elucidate biological themes that were in common or differed according to the treatment conditions. goSTAG is developed in R (open source) as an easy to use Bioconductor package and is publicly available at https://bioconductor.org/packages/goSTAG.

## Availability and requirements

- Project Name: goSTAG
- Project Home Page: The R Bioconductor package goSTAG is open source and available at https://bioconductor.org/packages/goSTAG
- Operating System: Platform independent
- Programming Language: R version ≥ 3.4.0
- License: GPL-3

## Additional file

Additional file 1: GMT file containing the gene symbols from the cancer therapeutics gene expression DEGs. (GMT 114 kb)

## Abbreviations

BP: Biological process
CC: Cellular component
Ctrl: Control
DEGs: Differentially expressed genes
FDR: False discovery rate
GEO: Gene Expression Omnibus
GMT: Gene matrix transposed
GO: Gene Ontology
goSTAG: GO subtrees to tag and annotate genes
MF: Molecular function
ORA: Over-representation analysis
Ox: Oxaliplatin
RNA-Seq: RNA sequencing
To: Topotecan
